# mm-Wave Substrate-Integrated Fabry–Perot/Leaky-Wave Antennas in E-Band

**DOI:** 10.3390/s25175248

**Published:** 2025-08-23

**Authors:** Rana Muhammad Hasan Bilal, Stefano Moscato, Simone Genovesi, Giuliano Manara, Filippo Costa

**Affiliations:** 1 Department of Information Engineering, University of Pisa, 56122 Pisa, Italy; rana.bilal@phd.unipi.it (R.M.H.B.); simone.genovesi@unipi.it (S.G.); giuliano.manara@unipi.it (G.M.); 2 Research and Development Department of SIAE MICROELETTRONICA, Via Michelangelo Buonarroti, 21, 20093 Cologno Monzese, Italy; stefano.moscato@siaemic.com

**Keywords:** Fabry–Perot, leaky-wave, high gain, PRS, mm-wave, E-band

## Abstract

This article introduces a substrate-integrated, low-cost, and low-profile E-band high-gain Fabry–Perot (FP)/leaky-wave (LW) antenna. This design enables the full integration of a high-gain antenna within a single-layer substrate for millimeter-wave (mm-wave) applications. The antenna design layout comprises a partially reflective surface (PRS) mounted on a thin, metal-coated, low-cost I-Tera MT40 dielectric substrate. The proposed antenna differs from conventional air-cavity-based FP/LW antennas, as it is fabricated on a low-cost dielectric substrate, eliminating the need for an air cavity, which restricts integration with printed circuit boards (PCBs) and planar circuits. The antenna is excited using a rectangular WR12 waveguide located beneath the ground plane. Impedance matching is achieved by employing a rectangular iris. The formulation for analyzing leaky waves within a cavity is thoroughly discussed using the Transverse Resonance Method (TRM). The proposed FP antenna achieves a maximum realized gain of 14.6 dBi with good impedance matching ($|S_{11}|$ = –14 dB). Finally, the proposed antenna is fabricated, and its performance is validated through experimental measurements.

## 1. Introduction

High-gain antennas are widely used in various applications, including satellite communications [1], automotive radar systems [2], indoor communications [3], astronomy [4], and mm-wave applications [5]. Cassegrain antennas are traditionally designed to achieve high gain, making them well-suited for long-range communication applications, such as satellite links, ground stations, and deep-space missions [6]. Although this approach can improve antenna gain, it presents several challenges, including complex feeding networks, large size, and intricate design topology [6]. To address these challenges, LWAs offer an efficient and promising solution, particularly for the mm-wave frequency range. They provide high gain (typically 10–20 dBi) with a single feed, feature a simple configuration, and are cost-effective to fabricate compared to conventional arrays and horn antennas [7].

Generally, an LWA consists of a simple structure: a single excitation source, typically a waveguide, and an air cavity between the ground plane and the radiating element [7]. This simple design makes it a suitable alternative to large horn antennas or complex feed arrays. Additionally, LWAs are designed in several ways, such as using electromagnetic band-gap (EBG) structures [8] or dielectric superstrates [9]. In recent years, the most effective approach to designing LWAs has involved the use of PRSs or FSSs. Trentini [10] initially introduced the PRS concept to achieve high antenna directivity by placing it above a ground plane. Subsequently, Jackson et al. investigated and advanced the gain-enhancement concept [11], introducing the term LWAs [12]. However, LWAs typically use an air spacer between the ground plane and the top PRS [13], with the cavity height selected to be half a wavelength at the operating frequency, which impedes their integration into microstrip circuits and other planar systems.

For example, a high-gain 60 GHz superstrate antenna was presented in [3]. This design used an aperture-coupled feeding mechanism and achieved a maximum gain of 14.6 dBi. Likewise, in [14], an air-filled cavity-based 94 GHz FP antenna was investigated, where a rectangular waveguide was used to excite the antenna cavity, achieving an overall gain of 25 dBi. However, many FP cavity-based antenna designs use air-filled cavities, which pose significant fabrication challenges, such as alignment issues, in addition to the associated assembly cost [15]. In [16], a 60 GHz FP antenna was explored, which employed a self-sustained grid that guarantees the presence of an air cavity between the feed and the grid.

The removal of the air cavity in the LWA is rarely addressed, as it typically decreases antenna performance due to substrate loss. However, substrate-integrated leaky antennas drastically reduce antenna cost, avoid alignment problems at mm-wave frequencies, and can be integrated into a single PCB and even on a chip. In recent years, a category of substrate-integrated leaky antennas has been explored specifically in the microwave range [17,18,19,20], but these configurations are typically one-dimensional transmission lines with two ports. The only attempt to design and fabricate a 2D mm-wave substrate-integrated LWA was presented in [21], where the authors achieved a gain of 16.7 dBi at 60 GHz. However, the proposed feeding mechanism, based on a coplanar slot in the ground plane, introduces a critical drawback: unwanted back radiation. A theoretical study on the effect of ohmic and dielectric losses in a dielectric-based cavity was presented in [22,23].

The objective of this paper is to further investigate the radiation properties of substrate-integrated LWAs operating at mm-wave frequencies. This is achieved by deriving closed-form expressions for the leaky-wave propagation constant, eliminating the need for a numerical solution of the dispersion equations. Additionally, we analyze the limitations of this approach compared to its air-cavity counterpart and highlight its practical advantages from a PCB-integration perspective. We also introduce a new design for a substrate-integrated LWA based on a waveguide feed. The performance of the antenna is analyzed both with and without the presence of the waveguide flange and alignment screws to verify its practical applicability. The proposed design offers a low-profile and highly cost-effective solution to synthesize a high-gain antenna in the E-band. Unlike conventional FPAs/LWAs that rely on air cavities requiring precise alignment, our antenna is fabricated in a single step using lithography, significantly reducing manufacturing complexity and cost. As noted in [23,24], alignment issues are particularly critical in the mm-wave and terahertz bands. To overcome these challenges, this work employs a printed antenna instead of an air-cavity structure. This approach ensures compatibility with PCBs and planar systems while eliminating the need for precise alignment mechanisms. Considering the standard cost associated with photolithography, the fabrication cost of this antenna is negligible relative to the overall system, making it a good choice for low-cost radar and Internet of Things (IoT) communication applications.

## 2. Design Layout of an E-Band LWA

The geometrical configuration of the analyzed LWA is inspired by inductive grids, selected for their simple shape and ease of fabrication. The LWA design consists of a standard three-layer configuration: a top PRS of inductive grids, a middle dielectric substrate, and a bottom ground plane.

**Figure 1:**
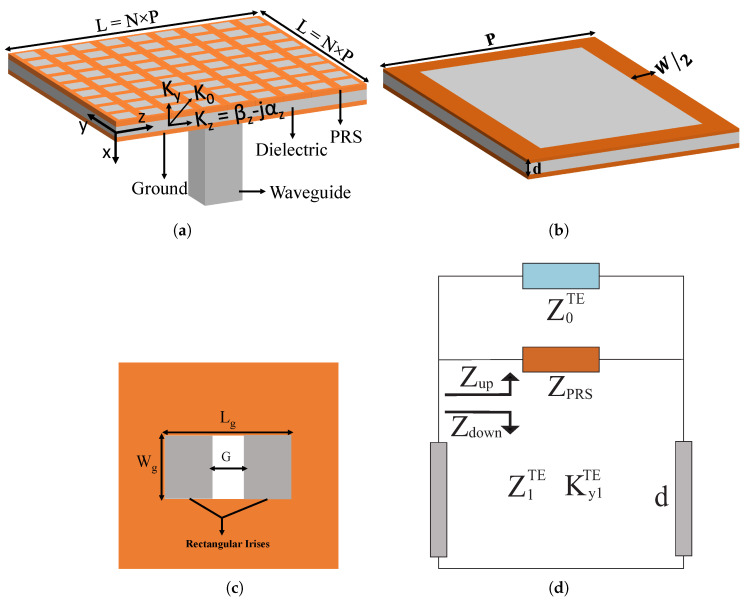
Proposed structure of the E-band LWA under analysis, with a total size (L×L) of 20.625mm×20.625mm: (**a**) complete antenna, (**b**) unit cell configuration, (**c**) back view showing inductive irises, and (**d**) transmission-line (TL) circuit model of the LWA.

The PRS on the top is printed on an I-Tera MT40 dielectric substrate by the Isola Group ($\varepsilon_{r}$ = 3.45; tanδ = 0.0027). The bottom layer is a slotted copper ground plane, with the slot included to facilitate excitation of the antenna through a rectangular waveguide. A pair of inductive rectangular irises is placed vertically at the location of the slot in the bottom ground plane to achieve impedance matching. The gap (G) between the two irises is varied to adjust the impedance matching of the antenna. All optimized geometric parameters are mentioned in the caption of Figure 1. The modeling and numerical simulation of the proposed LWA are performed using CST simulation software.

## 3. Leaky Analysis of the Antenna

The basic working principle of these antennas is based on the propagation of leaky waves, which are typically analyzed using the classical TRM [13,25]. The antenna dispersion equation is derived by applying the fundamental principle, which states that the sum of the upward and downward impedances must be zero, as given below in Equation (1):

(1) $$Z_{\mathrm{up}}+Z_{\mathrm{down}}=0$$

In Equation (1), $Z_{up}$ represents the input impedance above the dielectric substrate, and $Z_{down}$ represents the input impedance below the PRS. They are calculated using Equations (2) and (3):

(2) $$Z_{\mathrm{up}}=Z_{\mathrm{PRS}}\|Z_{0}^{TE/TM}$$

(3) $$Z_{\mathrm{down}}=jZ_{1}^{TE/TM}\tan\left(k_{y1}d\right)$$

where $Z_{0}^{\mathrm{TE}}=\frac{\omega \mu_{0}}{k_{y0}}$ and $Z_{0}^{\mathrm{TM}}=\frac{k_{y0}}{\omega \varepsilon_{0}}$, and $Z_{1}^{\mathrm{TE}}=\frac{\omega \mu_{0}}{k_{y1}}$ and $Z_{1}^{\mathrm{TM}}=\frac{k_{y1}}{\omega \varepsilon_{0}\varepsilon_{r}}$, with $k_{y0}=\sqrt{k_{0}^{2}-k_{z}^{2}}$ and $k_{y1}=\sqrt{k_{0}^{2}\varepsilon_{1}-k_{z}^{2}}$.

The top PRS consists of periodic arrays of inductive grids, and its impedance can be calculated using the following Equation (4) [26]:

(4) $$Z_{PRS}=R_{PRS}+j\frac{\omega \mu_{0}P}{2\pi }\ln\left(\frac{1}{\sin\left(\frac{\pi W}{2P}\right)}\right)$$

**Figure 2:**
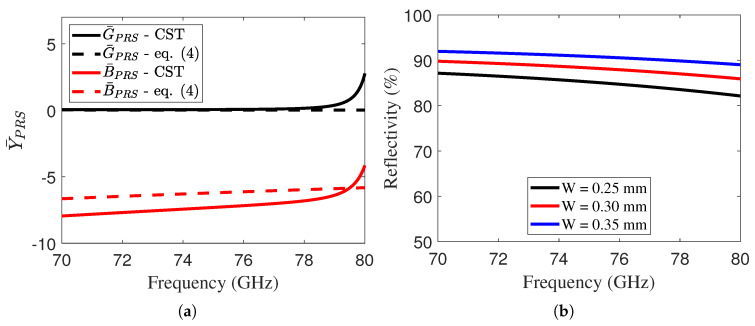
(**a**) Analytical and CST-simulated admittance trend of the PRS unit cell, and (**b**) reflectivity trend of the unit cell for varying *W*. The unit cell is shown in Figure 1b.

In the above equation, *P* represents the periodicity of the inductive grid, *W* denotes the width of the grid, and $\mu_{0}$ is the permeability of the free space. The grid impedance can also be defined in terms of admittance as $Y_{PRS}=\frac{1}{Z_{PRS}}=G_{PRS}+jB_{PRS}$. In Figure 2a, we report the normalized PRS admittance ${\bar{Y}}_{PRS}=Y_{PRS}\eta_{0}$ with $\eta_{0}=\sqrt{\frac{\mu_{0}}{\varepsilon_{0}}}$. The behavior obtained from the CST simulations is compared with the results derived from Equation (4). The PRS impedance is extracted from full-wave simulations by analyzing a single unit cell of the structure and applying transmission-line inversion techniques [27]. Moreover, the reflectivity of the unit cell is presented in Figure 2b. By solving Equation (1) using a similar approach to that in [13,28], we obtain Equation (5):

(5) $$k_{y1}^{\mathrm{TE}/\mathrm{TM}}=j\frac{Z_{\mathrm{up}}}{dZ_{1}^{\mathrm{TE}/\mathrm{TM}}}+\frac{\pi }{d}$$

By substituting Equation (2) and $Z_{1}^{TE}$ into Equation (5), a closed-form expression for the TE propagation constant can be obtained, as given in Equations (6) and (7):

(6) $$k_{y1}^{\mathrm{TE}}=\frac{\pi \omega \mu_{0}}{\omega \mu_{0}d-jZ_{\mathrm{up}}}$$

(7) $$k_{z}^{\mathrm{TE}}=\sqrt{k_{0}^{2}\varepsilon_{1}-{\left(k_{y1}^{\mathrm{TE}}\right)}^{2}}$$

**Figure 3:**
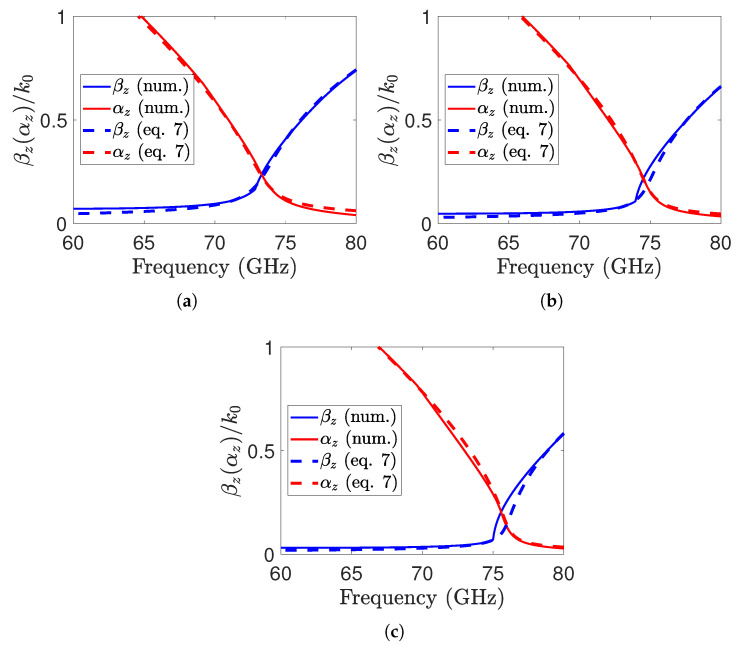
Complex leaky-wave propagation constant $k_{z}^{TE}=\beta_{z}-j\alpha_{Z}$ of the LWA on the I-Tera MT40 ($\varepsilon_{r}=3.45$) with d=1 mm. The numerical solution, obtained by solving Equation (1), is compared with the closed-form Equation (7). The results are shown for P=0.825 mm and different grid widths: (**a**) W=0.25 mm, (**b**) W=0.30 mm, and (**c**) W=0.35 mm.

The closed-form expression derived from the leaky-wave propagation constant TE is compared with the numerical solution in Figure 3a for W=0.25mm. It is evident that the proposed analytic solution is extremely accurate, as it aligns well with the numerical one. The working frequency (broadside radiation) of the LWA is expected to be at the frequency corresponding to the splitting condition [25], which is affected by the reflectivity of the PRS. Furthermore, the leaky-wave propagation constant is calculated for larger grid widths, specifically W=0.3mm and W=0.35mm, as shown in Figure 3b,c. It is observed that increasing *W* shifts the operating frequency towards higher values.

Although air-cavity and low-loss materials are well established as preferred choices to maximize directivity and bandwidth in LWAs [29], this work explores a different approach by employing a lossy dielectric substrate in the design of an LWA [22,23]. According to [29], the directivity–bandwidth figure of merit (FoM) is inversely proportional to the relative permittivity given in Equation (8):

(8) $$\mathrm{FoM}=D\cdot \mathrm{FBW}\approx \frac{2.47}{\varepsilon_{r}}$$

where *D* represents the directivity of the antenna and FBW denotes the fractional bandwidth. According to Equation (8), for a fixed FBW, the antenna directivity is expected to decrease as the dielectric permittivity of the substrate increases. In this study, even though the substrate is a lossy substrate, effective leaky-wave radiation is still ensured. The optimal normalized attenuation constant, $\hat{\alpha }=\alpha /k_{0}$, accounting for both substrate and grid losses, can be computed following the approach given in Equation (9) [22]:

(9) $$\begin{array}{cc} \; & \hat{\alpha }≃\sqrt{\frac{\sqrt{\varepsilon_{r}^{3}}}{\pi }\frac{1+{\bar{G}}_{\mathrm{PRS}}}{{\left(1+{\bar{G}}_{\mathrm{PRS}}\right)}^{2}+{\bar{B}}_{\mathrm{PRS}}^{2}}+\frac{\varepsilon_{r}\tan\delta }{2}}\\ \; \end{array}$$

where ${\bar{G}}_{PRS}=G_{PRS}\eta_{0}$. The efficiency of the lossy antenna can be estimated as given in Equation (10) [22]:

(10) $$\eta =\eta_{L}\frac{{\left.\hat{\alpha }\right|}_{{\bar{G}}_{\mathrm{PRS}}=0,\tan\delta =0}^{2}}{{\hat{\alpha }}^{2}}$$

where $\eta_{L}=1-\exp\left(\frac{-2\pi \hat{\alpha }L}{\lambda_{0}}\right)$. For our antenna, the optimal normalized attenuation constant $\hat{\alpha }$ is found to be 0.21, which closely matches the estimated value in Figure 3a. Furthermore, based on Equation (10), the estimated efficiency of the proposed design is 0.84, with $\eta_{L}$ approaching 1.

## 4. Results and Discussion

The fundamental step in designing an LWA is the choice of a unit cell capable of achieving high reflectivity (R > 90%) to ensure high directivity, as described by $D_{\mathrm{Max}}\approx \frac{1+R}{1-R}$ [30]. For this purpose, an inductive grid backed by a dielectric substrate was selected, as illustrated in Figure 1b. The unit cell is simulated with unit cell boundary conditions along the x and y directions, and open add-space boundaries along the z direction. Figure 2b illustrates the reflectivity trend as a function of the grid width *W*. It is important to emphasize that the larger the *W*, the higher the reflectivity, which consequently leads to a higher quality factor and a potential increase in directivity [13]. However, in this design, because the cavity is dielectric, the increase in the quality factor may result in an increase in losses. In this work, the periodicity of the unit cells is set to 0.825 mm, while the antenna operates at 73.5 GHz, which corresponds to a wavelength ($\lambda_{0}$) of 4 mm in free space. Considering the dielectric substrate with an $\epsilon_{r}$ of 3.45, the guided wavelength ($\lambda_{g}$) is approximately 2.15 mm. Since the chosen periodicity (P=0.825 mm) is significantly smaller than $\lambda_{g}$, the structure operates well below the Bragg condition. This ensures that Bragg modes are not excited within the operating frequency range.

**Figure 4:**
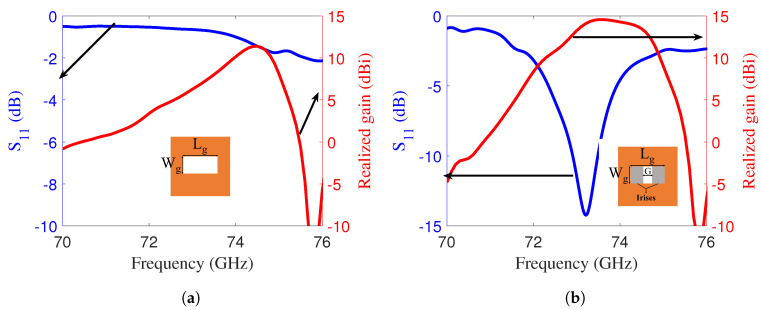
(**a**) $|S_{11}|$ and realized gain of the E-band LWA with an open waveguide; the inset shows the antenna back view. (**b**) $|S_{11}|$ and realized gain of the E-band LWA with metallic strips; the inset shows the antenna back view.

The complete configuration of the proposed LWA, with overall dimensions of 20.625mm×20.625mm, was designed to optimize key performance metrics, including the gain and the reflection coefficient ($|S_{11}|$). The selected antenna size was based on the requirement to excite it using a WR12 waveguide, which has a circular flange diameter of 19 mm. To ensure proper alignment and mounting, the antenna was designed slightly larger than the waveguide flange, allowing for secure and efficient integration. Firstly, it was noted that the antenna demonstrates poor impedance matching, as indicated by an $|S_{11}|$ value of approximately –1 dB at 73.5 GHz. Furthermore, it achieves a realized gain of only 6 dBi at the same frequency (Figure 4a) due to poor impedance matching. To achieve a better match, a pair of rectangular strips was introduced within the slot of the ground plane, as shown in Figure 1c. Before introducing inductive irises, the antenna exhibited poor impedance matching that severely reduced radiation efficiency and, consequently, the total efficiency, even though the intrinsic radiation efficiency of the radiating structure was acceptable. The inductive irises were employed primarily to control the phase and impedance along the waveguide, thereby improving impedance matching over the operating band. By reducing the input reflection, the radiation efficiency increases, which directly raises the total efficiency.

**Figure 5:**
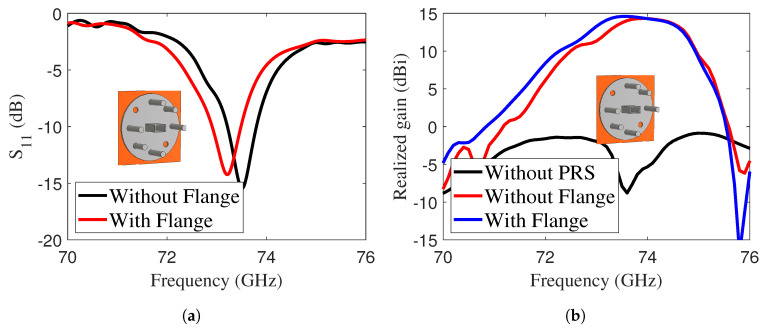
(**a**) $|S_{11}|$ of the LWA with and without mechanical attachment of the waveguide flange, and (**b**) realized gain of the LWA with and without mechanical attachment of the waveguide flange.

The width of the iris, denoted *G*, serves as a key parameter to adjust the magnitude of the $|S_{11}|$ response. As shown in Figure 4b, the antenna achieved an improved $|S_{11}|$ value of –14 dB, accompanied by a corresponding realized gain of approximately 14.6 dBi. The effect of flanges and alignment screws was also analyzed to verify the performance of the designed antenna with a real E-band waveguide. The waveguide flange is made of copper, modeled as a perfect electric conductor (PEC) in the simulation. Its electrical conductivity is $\sigma =5.8\times 10^{7}\;S/m$. Figure 5a,b compare $|S_{11}|$ and the realized gain of the LWA with and without the waveguide flange attachment, shown in the inset. The realized gain remained unchanged, with a slight shift observed in $|S_{11}|$.

**Figure 6:**
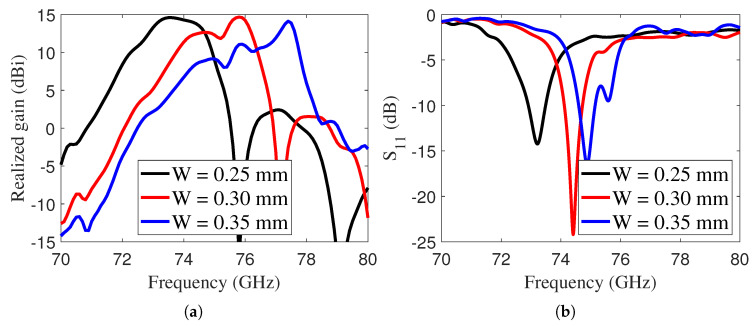
(**a**) Realized gain of the E-band LWA with varying *W*, and (**b**) $|S_{11}|$ of the E-band LWA with varying *W*.

Moreover, the realized gain and $|S_{11}|$ of the LWA were analyzed across varying widths of the inductive grid, as illustrated in Figure 6. It should be noted that the width of the grid plays a significant role in controlling the antenna gain. A large grid width enhances reflectivity, thereby increasing the gain; however, this comes at the cost of reduced bandwidth.

**Figure 7:**
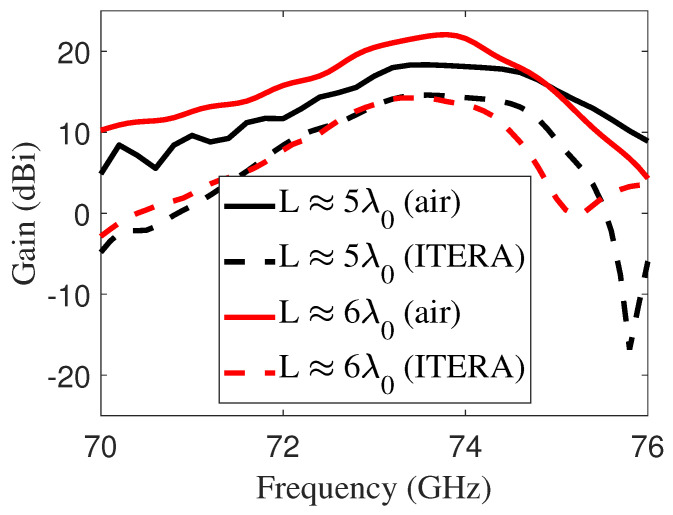
Gains for two antenna configurations (conventional and proposed) at two antenna sizes, where $\lambda_{0}=4$ mm at an operating frequency of 73.5 GHz. The solid line represents the conventional FPA with an air cavity of height $\lambda_{0}/2$ (2 mm), while the dashed line corresponds to the proposed substrate-integrated FPA, which employs a low-cost I-Tera MT40 dielectric substrate with a thickness of d=1 mm.

Furthermore, Figure 6b illustrates the antenna performance $|S_{11}|$ for varying grid widths, showing that the resonance frequency of the LWA shifted to higher values as the grid width increased. The gain of the LWA was analyzed for two antenna sizes, as shown in Figure 7. To highlight the difference from the more classic air-cavity design, a 2 mm thick air-cavity antenna was also introduced. It achieved peak gains of 18.20 dBi and 22 dBi at 73.5 GHz for sizes of $5\lambda_{0}$ and $6\lambda_{0}$, respectively. The peak gain dropped to 14.6 dBi when using the I-Tera MT40 substrate due to dielectric losses. These losses are not present in designs that utilize air cavities, which helps maintain higher gain levels.

**Figure 8:**
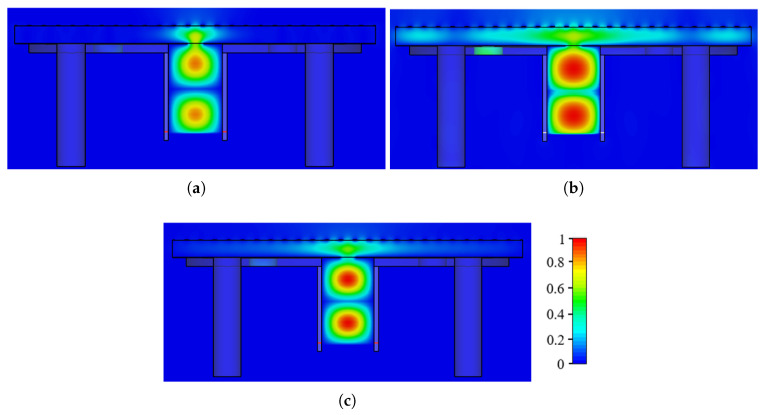
Color plots of the electric field distribution inside the FP cavity on the xz plane at (**a**) 71 GHz, (**b**) 73.5 GHz, and (**c**) 75.5 GHz.

This section analyzes the electric field distribution inside the FP cavity to assess how well the antenna is illuminated. Three frequency points are considered: 71 and 75.5 GHz, where the antenna gain is zero due to partial cavity illumination, and 73.5 GHz, where the gain is maximized, indicating full cavity excitation. Figure 8 illustrates the electric field distribution at different operating frequencies. At 71 GHz, the cavity remains unilluminated, resulting in zero gain (see Figure 8a). In contrast, at 73.5 GHz, most of the cavity is excited, achieving a gain of approximately 14.6 dBi (see Figure 8b). Similarly, at 75.5 GHz, the cavity is not excited, leading to minimal gain (see Figure 8c).

**Figure 9:**
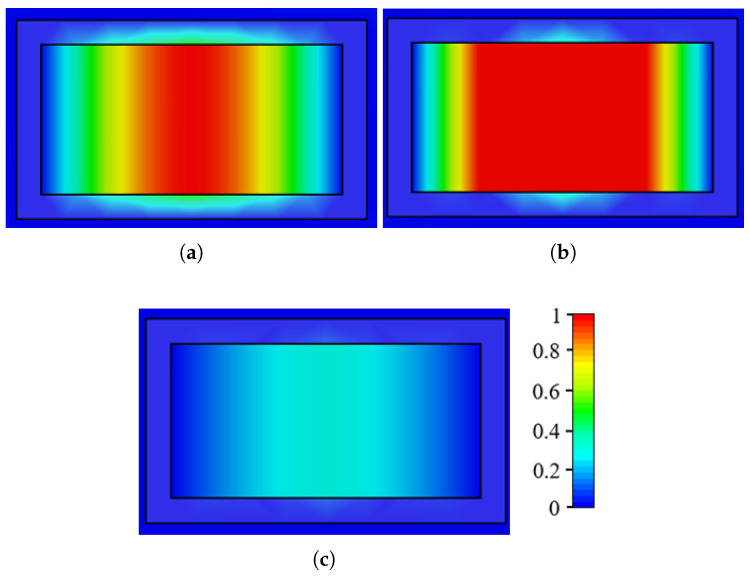
Electric field distribution within the slotted ground plane and iris region at (**a**) 71 GHz, (**b**) 73.5 GHz, and (**c**) 75.5 GHz.

Additionally, Figure 9 illustrates the normalized electric field distribution within the slotted ground plane and the iris region at three different frequencies: 71 GHz, 73.5 GHz and 75.5 GHz. At 71 GHz (Figure 9a), the field is strongly concentrated in the central portion of the slot, gradually decaying toward the edges. At 73.5 GHz (Figure 9b), the electric field reaches its maximum intensity throughout the central area, indicating strong resonance and efficient confinement of energy. In contrast, at 75.5 GHz (Figure 9c), the field magnitude decreases significantly, showing weaker confinement and energy coupling in the region.

**Figure 10:**
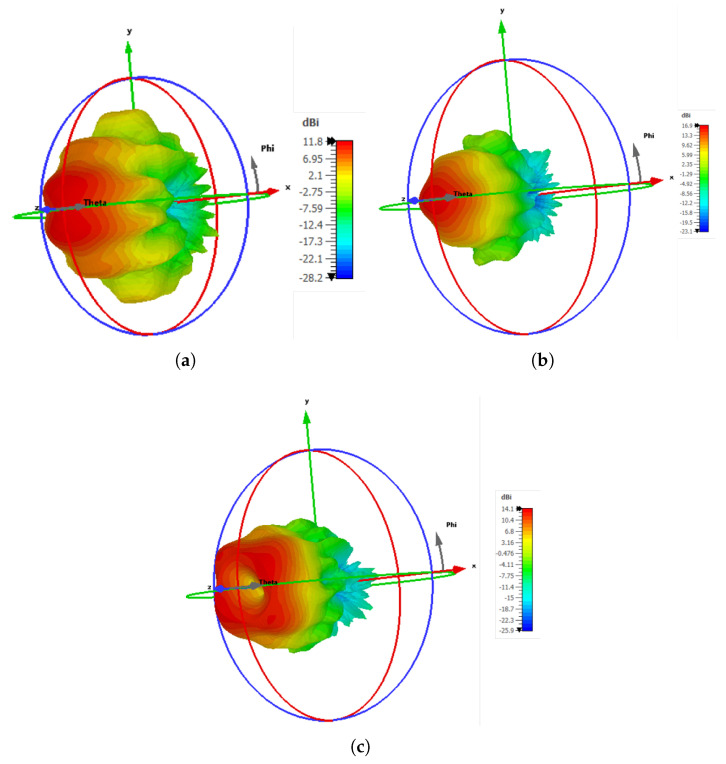
Three-dimensional radiation patterns of the E-band LWA a t (**a**) 71 GHz, (**b**) 73.5 GHz, and (**c**) 75.5 GHz.

Furthermore, the 3D far-field radiation pattern of the proposed antenna is analyzed for three different operating frequencies, as shown in Figure 10. Initially, the 3D far-field radiation pattern of the proposed LWA at 71 GHz exhibits a highly directional main beam with a peak directivity of approximately 11.8 dBi, as presented in Figure 10a. This high directivity is attributed to constructive interference within the cavity formed between the ground and the PRS, which supports a fast leaky mode. The antenna shows a side-lobe level (SLL) of approximately –5.8 dB, which is typical for uniformly excited apertures without amplitude tapering. At 75.5 GHz, the antenna achieves a maximum directivity of 14.07 dBi, with a null in the broad side direction ($\theta =0^{\circ }$), resulting in a distinct hole in the center of the main beam, as illustrated in Figure 10c. Likewise, at 73.5 GHz, the antenna exhibits a high directivity of 16.99 dBi, confirming the formation of a well-defined main beam oriented towards the broadside, as shown in Figure 10b. The SLLs are lower than the main lobe, indicating satisfactory radiation performance.

## 5. Experimental Validation

**Figure 11:**
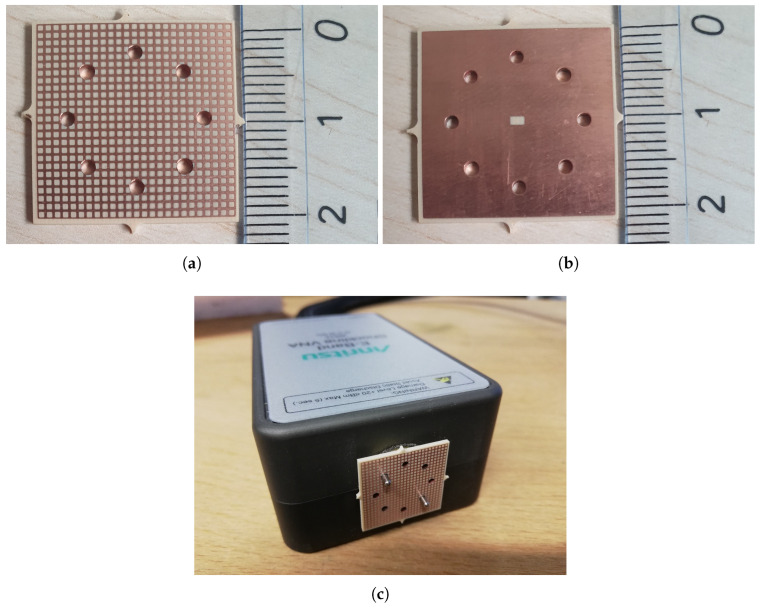
Fabricated prototype of the E-band LWA: (**a**) top antenna structure; (**b**) back view of the antenna, showing the alignment holes and the slot in the ground plane; and (**c**) antenna sample with the E-band VNA extender module.

The proposed antenna was fabricated using a standard photolithographic process on an I-Tera MT40 dielectric substrate with a thickness of *d* = 1 mm, as shown in Figure 11. Figure 11a presents the top view of the fabricated antenna, featuring a 25×25 array of inductive grids with an overall size of 20.625mm×20.625mm. Similarly, Figure 11b shows the back view of the antenna, which includes a slotted ground plane with multiple holes designed to align the waveguide to excite the antenna. Finally, Figure 11c illustrates the measurement setup, where the antenna sample is connected to the E-band VNA module.

**Figure 12:**
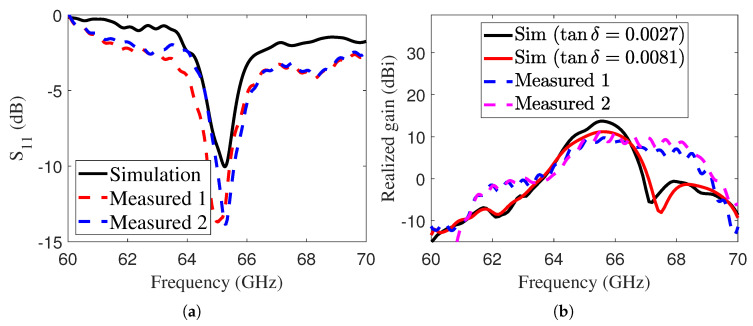
Simulated and measured (**a**) $|S_{11}|$ and (**b**) gain of the E-band LWA. Measurements on two different prototypes are reported to verify the replicability and fabrication tolerances.

To validate the simulated performance, we performed several measurements to verify the simulation results, but only two are shown here. During testing, we found that the dielectric did not behave exactly as described in the datasheet. After fabricating and measuring several antenna samples, we consistently observed the reflection dip at around 65 GHz, while the simulation showed it at 73.5 GHz. To make a fair comparison, we used adjusted values that matched the experimental results more closely. According to the datasheet, the I-Tera MT40 dielectric substrate has electrical properties of $\varepsilon_{r}=3.45$ and tanδ=0.0027. However, these properties are measured out-of-plane. Since the FP antenna exploits the TE01 mode [31] of the cavity, we are more interested in the in-plane properties of the material, measured parallel to the substrate surface. In this case, the supplier specifies $\varepsilon_{r}=3.72$ and tanδ=0.007 at 70 GHz. In our simulations, to best fit the experimental data, $\varepsilon_{r}$ was adjusted to 3.9 and tanδ to 0.0081. The $|S_{11}|$ and realized gain of the LWA were measured and compared with the simulated results, as illustrated in Figure 12a,b. The realized gain was simulated for two different values of tanδ: 0.0027 and 0.0081. Although the simulated gain with tanδ=0.0027 was higher, the trend remained consistent with the measured data. The discrepancy between the simulated and measured gains is attributed to the properties of the substrate. In order to verify the influence of fabrication tolerances, two identical prototypes were fabricated and measured. The two prototypes exhibit very similar properties, as shown in Figure 12.

**Figure 13:**
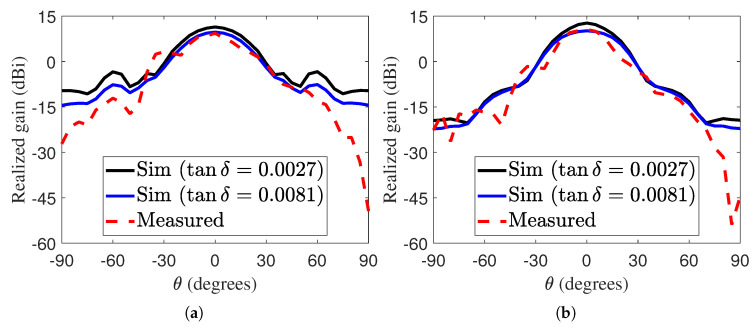
Simulated and measured radiation patterns of the proposed LWA at (**a**) 65 GHz and (**b**) 66 GHz.

In this section, we used $\varepsilon_{r}=3.9$ again to calculate the radiation pattern, as it is the predicted value of the dielectric substrate. Figure 13 shows the radiation patterns of the LWA at two operating frequencies: 65 GHz and 66 GHz. As shown in Figure 13, the measured and simulated radiation patterns are in close agreement for both operating frequencies, with the main beam consistently directed toward the boresight. Likewise, results are reported for two values of tanδ to show that it impacts only the peak gain, but not the shape of the radiation pattern or –3 dB angles.

At high frequencies, small geometric or material deviations can affect beam direction and impedance matching, so we used a high-precision fabrication method to meet the simulated design parameters. The antenna performance is sensitive to the dielectric constant and the thickness of the substrate. Variations in substrate permittivity can change the angle of the beam or alter impedance matching. To mitigate this, we used a highly stable, low-loss substrate (the I-Tera MT40), which exhibits minimal variation and is suitable for high-frequency applications. Additionally, for waveguide-fed LWAs, precise alignment between the source and the antenna is essential to ensure consistent excitation and minimize reflection. Misalignment may lead to beam-pointing errors or cause poor impedance matching or degraded gain. Moreover, at high frequencies such as the mm-wave band, thermal effects can affect antenna performance as a result of localized heating, which may cause slight variations in the dielectric permittivity and potentially affect beam direction and impedance matching in high-frequency applications.

Table 1 presents a comparison of existing LWAs designed for the E-band spectrum, highlighting their design methods, feeding mechanisms, and achieved gains. The comparison is limited to narrowband mm-wave LWAs to underscore the simplicity of the proposed antenna, which employs a single-layer structure without the need for an air cavity. Unlike the reported works that rely on air-spacer cavities, the proposed LWA is fabricated on an I-Tera MT40 dielectric substrate, which simplifies the fabrication process. In addition, it utilizes simple waveguide excitation for feeding, unlike the reported designs that rely on complex feeding networks, making fabrication more challenging and costly.

## 6. Conclusions

A single-layer mm-wave substrate-integrated LWA operating in the E-band was designed and tested. The LWA comprised a single dielectric block (I-Tera MT40) with a 25×25 array of inductive-grid unit cells printed on the top side and a slotted ground plane on the bottom surface. This antenna differed from previously reported mm-wave LWAs, which used an air-spacer cavity rather than a dielectric substrate. Two metallic strips were vertically placed and optimized to achieve good impedance matching. A leaky-wave analysis of the dielectric cavity was performed by deriving a new closed-form expression for the TE propagation constant. The performance of the antenna was evaluated by comparing the results of full-wave simulations with the measured data from multiple prototypes. 

## Figures and Tables

**Table 1:** Performance analysis of existing mm-wave FPAs/LWAs compared with the current study.

Design	Feeding	Gain (dBi)	−10 dB Bandwidth (%)	Size ($\lambda_{0}^{2}$)	Thickness ($\lambda_{0}$)	Fabrication
Multi-layer [3]	Coax + patch	14.6	6.5	6×6	0.69	Photolithography and etching
Multi-layer [14]	Waveguide	25	2.6	11×11	0.67	N/A
Multi-layer [15]	Coax + slot	16	18.4	3×3.6	1.13	PCB
Single layer [16]	Coax + slot	13	5.54	4.4×4.4	0.51	Photolithography
Single layer [21]	Co-planar slot	16.7	2.35	6×6	0.47	Double-sided photolithography
Multi-layer [32]	Coax + patch	17.7	12.90	5×5	0.70	PCB
Single layer (proposed)	Waveguide	14.6	0.55	5×5	0.5	Photolithography

## Data Availability

Data will be made available on reasonable request from the authors.
